# High-throughput screening against thioredoxin glutathione reductase identifies novel inhibitors with potential therapeutic value for schistosomiasis

**DOI:** 10.1186/s40249-015-0071-z

**Published:** 2015-08-31

**Authors:** Ting Li, Peter D. Ziniel, Pan-qing He, Valerie P. Kommer, Gregory J. Crowther, Min He, Qing Liu, Wesley C. Van Voorhis, David L. Williams, Ming-Wei Wang

**Affiliations:** The National Center for Drug Screening, Shanghai Institute of Materia Medica, Chinese Academy of Sciences, Shanghai, 201203 China; Department of Immunology and Microbiology, Rush University Medical Center, Chicago, IL 60612 USA; Division of Allergy and Infectious Diseases, University of Washington, Seattle, WA 98195 USA

**Keywords:** High-throughput screening, Schistosomiasis, Inhibitor, Thioredoxin glutathione reductase

## Abstract

**Background:**

Schistosomiasis, a parasitic disease also known as bilharzia and snail fever, is caused by different species of flatworms, such as *Schistosoma mansoni* (*S. mansoni*). Thioredoxin glutathione reductase (TGR) from *S. mansoni* (SmTGR) is a well-characterized drug target for schistosomiasis, yet no anti-SmTGR compounds have reached clinical trials, suggesting that therapeutic development against schistosomiasis might benefit from additional scaffolds targeting this enzyme.

**Methods:**

A high-throughput screening (HTS) assay *in vitro* against SmTGR was developed and applied to a diverse compound library. SmTGR activity was quantified with ThioGlo®, a reagent that fluoresces upon binding to the free sulfhydryl groups of the reaction product GSH (reduced glutathione).

**Results:**

We implemented an HTS effort against 59,360 synthetic compounds. In the primary screening, initial hits (928 or 1.56 %) showing greater than 90 % inhibition on SmTGR activity at a final concentration of 10 μM for each compound were identified. Further tests were carried out to confirm the effects of these hits and to explore the concentration-dependent response characteristics. As a result, 74 of them (0.12 %) representing 17 chemical scaffolds were confirmed and showed a great concentration-dependent inhibitory trend against SmTGR, including structures previously shown to be lethal to schistosomal growth. Of these, two scaffolds displayed a limited structure-activity relationship. When tested in cultured larvae, 39 compounds had cidal activity in 48 h, and five of them killed larvae completely at 3.125 μM. Of these, three compounds also killed adult worms *ex vivo* at concentrations between 5 μM and 10 μM.

**Conclusion:**

These confirmed hits may serve as starting points for the development of novel therapeutics to combat schistosomiasis.

## Background

Schistosome parasites infect an estimated 200 million people, of which 20 million have significant morbidity, resulting in more than 200,000 deaths annually. It is estimated that nearly 800 million people are at risk of infection [1–3]. At present, schistosomiasis control strategies rely almost exclusively on chemotherapy; tens of millions of people are treated with the only available drug, praziquantel (PZQ) [4, 5]. With the current levels of drug use, it is inevitable that PZQ-resistant parasites will evolve. Since no new drugs are in the clinical pipeline to replace it, it is imperative to identify new targets and drug candidates for schistosomiasis treatment.

Mechanisms to maintain redox balance have been shown to be essential for schistosome worm survival. Previous studies have found significant differences between the redox network of schistosomes and their human hosts, with worm defenses being significantly less robust [6–9]. An important chokepoint in the redox network is thioredoxin glutathione reductase (TGR), which replaces three enzyme activities found in humans: glutathione reductase, thioredoxin reductase, and glutaredoxin (deglutathionylation) [8, 9]. Using RNAi (RNA interference) and chemical probes, TGR has been shown to be an essential worm protein and a druggable target. A screen against the schistosome redox pathway of TGR and peroxiredoxin-2 identified oxadiazole-2-oxides as novel TGR inhibitors with significant *in vivo* activity against laboratory infections [10–13]. Here we present results from a new high-throughput screening (HTS) effort against *Schistosoma mansoni* (*S. mansoni*) TGR (SmTGR). Several novel compounds have been discovered with activity against SmTGR, cultured larval, and adult worms. These compounds may serve as starting points for the development of new therapeutic agents to combat schistosomiasis.

## Methods

### Reagents

Thioredoxin glutathione reductase (EC 1.8.1.B1) from *S. mansoni* (SmTGR) was produced at the laboratory of Dr. Wesley Van Voorhis (University of Washington, Seattle, WA, USA) by methods described previously [8]. Naphthazarin, oxidized glutathione (GSSG), β-nicotinamide adenine dinucleotide 2’-phosphate reduced tetrasodium salt hydrate (NADPH), and ethylenediaminetetraacetic acid (EDTA) were supplied by Sigma-Aldrich (St. Louis, MO, USA). Bovine serum albumin (BSA) was the product of AMRESCO (Solon, OH, USA). ThioGlo® 1 Fluorescent Thiol Reagent was obtained from Merck (Darmstadt, Germany).

### Endpoint SmTGR assay

SmTGR protein (1.58 μg/ml) was loaded into each well of black 384-well plates (PerkinElmer, Boston, MA, USA) containing 19 μl of assay buffer I (90 mM Tris-HCl, 10 mM EDTA, pH 7.5, 100 μM NADPH, and 0.48 mg/ml BSA), followed by an addition of 1 μl positive control or test compounds. The plates were sealed and incubated for 50 min at room temperature. Then, 10 μl of assay buffer II (90 mM Tris-HCl, 10 mM EDTA, pH 7.5, and 100 μM NADPH) supplemented with 150 μM GSSG was added to give a final volume of 30 μl per well. Following a 1 h incubation, the product was reacted with 30 μl ThioGlo® 1 reagent for 20–30 min, and the fluorescence signal was monitored at an excitation wavelength of 380 nm and an emission wavelength of 515 nm on the EnVision plate reader (PerkinElmer).

### Compound library

The compound library comprising 59,360 synthetic compounds was provided by Novo Nordisk A/S (Bagsværd, Denmark). The structural diversity covers heterocycles, lactams, sulfonates, sulfonamides, amines, secondary amides, and natural product-derived compounds. The compounds were highly purified and the stock, pre-dissolved in 100 % dimethyl sulfoxide (DMSO) solution, was applied to the primary screening with an average final concentration of 10 μM for each compound.

### High-throughput screening experiment

An HTS effort was carried out against the compound library described above. In each of the 384-well plates, 64 wells of the outer four columns were used as high-, mid-, and low-concentration of naphthazarin controls (final concentrations: 30 μM, 1.5 μM, and 0.5 μM, respectively) and negative control (3.3 % DMSO), each with 16 replicates. The test compounds were placed in the center columns 3–22. Both uninhibited (DMSO) and fully inhibited (30 μM naphthazarin) signals were assessed and the Z' factor was calculated according to the literature [14]. Taking 30 μM naphthazarin to represent 100 % inhibition, compounds showing greater than 90 % inhibition were considered as ‘hits’. All initial hits were rescreened and further studied for concentration-dependent response characteristics.

### Studies with parasites

Cercariae were shed from infected *Biomphalaria glabrata* snails, obtained from the Biomedical Research Institute (Rockville, MD, USA), and mechanically transformed to schistosomula as described [15]. Approximately 300 freshly prepared schistosomula were placed in each well of a 24-well plate containing 1 ml Basch’s Complete Medium 169 (with the addition of 10 % fetal bovine serum) and incubated overnight at 37 °C in 5 % CO_2_ atmosphere. The following day, compounds were added to each well at the indicated concentrations and the parasites were observed for several days for dead (dark, granular appearance, and non-motile) or alive (translucent and motile) as described [16]. Adult parasites were obtained from female Swiss Webster mice seven weeks after infection by perfusion with RPMI Medium 1640 (Thermo Fisher Scientific, Waltham, MA, USA) using standard methods [15]. Live worms were washed thoroughly with Dulbecco’s Modified Eagle’s Medium (Life Technologies, Carlsbad, CA, USA), and incubated in 5 ml Basch’s Complete Medium 169 [17] in 6-well tissue culture plates with ten worm pairs per well, and cultured overnight in 5 % CO_2_ at 37 °C. The following day, media were removed from each well and replaced with 5 ml of fresh media. Test compounds were dissolved in DMSO at 10 mM and added to the wells at the indicated concentrations. The same volume of DMSO was added to each well. Negative control worms were treated with an equal volume of DMSO alone. Each well was replaced with fresh media and compounds every 48 h. Worm mobility and survival were observed under a Zeiss Stemi 2000-C stereomicroscope (Carl Zeiss, Jena, Germany) for 10 sec per worm. This study was approved by the Institutional Animal Care and Use Committee at Rush University Medical Center (IACUC number 14–080; DHHS animal welfare assurance number A3120-01).

### Data analysis

Data were analyzed using GraphPad Prism® software (GraphPad, San Diego, CA, USA). Nonlinear regression analyses were performed to calculate IC_50_ values. Values presented are means ± standard error of the mean (SEM) of at least three independent experiments. Percentage inhibition of SmTGR activity by hit compounds was calculated after defining the response to 30 μM naphthazarin as 100 % inhibition.

## Results

### Assay optimization and validation

Recombinant SmTGR was expressed and purified as previously described [8]. An earlier assay of the coupled activities of SmTGR and peroxiredoxin-2 [13] was adapted to study the activity of SmTGR alone, using the following principle: SmTGR converts NADPH and GSSG to NADP^+^ and reduced glutathione (GSH), and GSH production can be detected with ThioGlo®, a maleimide derivative of the naphthopyranone fluorophore which fluoresces upon reacting with free thiol groups such as those present in GSH. We performed the assay in 384-well plates with a reaction volume of 30 μl before addition of ThioGlo®. Final concentrations prior to ThioGlo® addition were 1 μg/ml SmTGR, 100 μM NADPH, and 50 μM GSSG in a buffer of 90 mM Tris-HCl, pH 7.5, with 10 mM EDTA and 0.45 mg/ml BSA. After 60 min of room-temperature incubation, the reaction was stopped with 30 μl of 20 μM ThioGlo 1 (Covalent Associates, Corvallis, OR, USA). After ten additional minutes, samples were excited at 380 nM and read for fluorescence at 515 nM.

### High-throughput screening parameters and execution

**Fig. 1:**
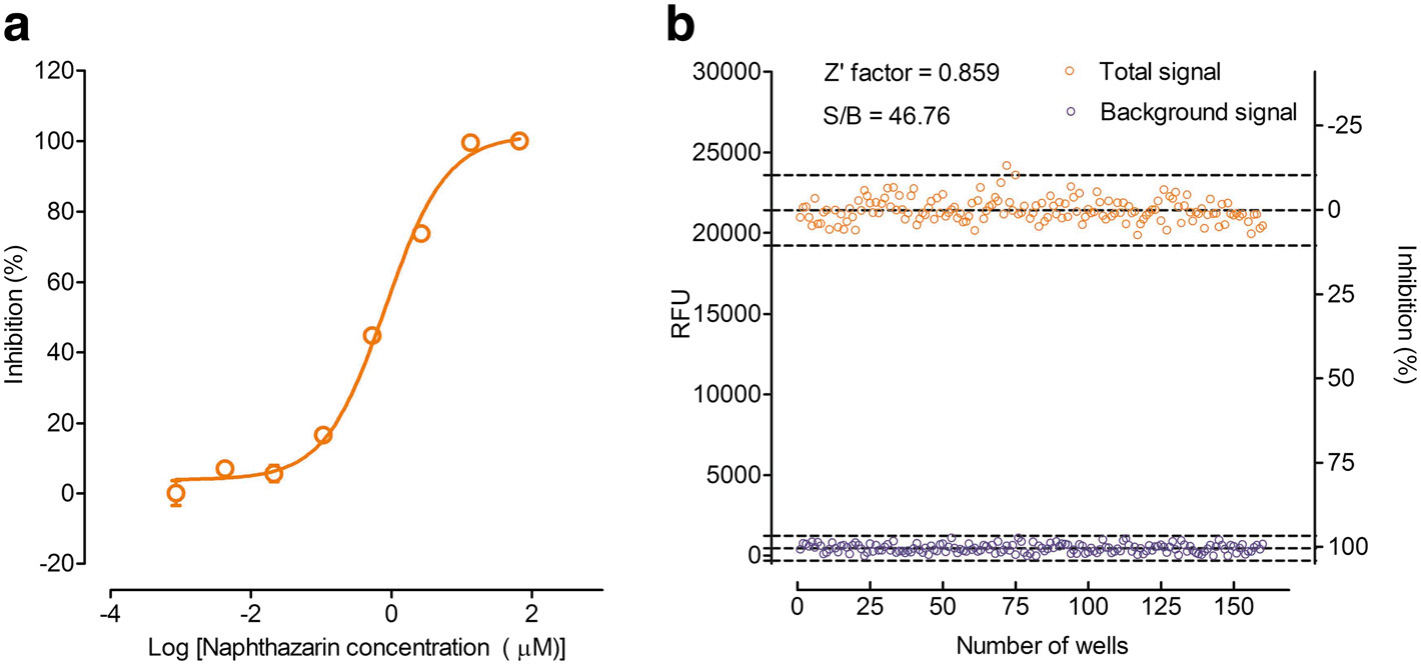
Validation of the HTS assay. **a** Concentration-dependent inhibitory activity of naphthazarin on SmTGR under the optimized assay conditions, from which the IC_50_ value was calculated (*n* = 3, mean ± SEM). **b** Z' factor determination. Assays were performed under the optimized conditions and 160 replicates of total and background signals were studied. HTS assay parameters, including Z' factor and S/B were examined. Raw fluorescence units (Y-axis in the left) from each of the controls and % inhibition (Y-axis in the right) are shown. Dashed lines indicate means and mean ± 3 × standard deviation of the 160 data points

**Fig. 2:**
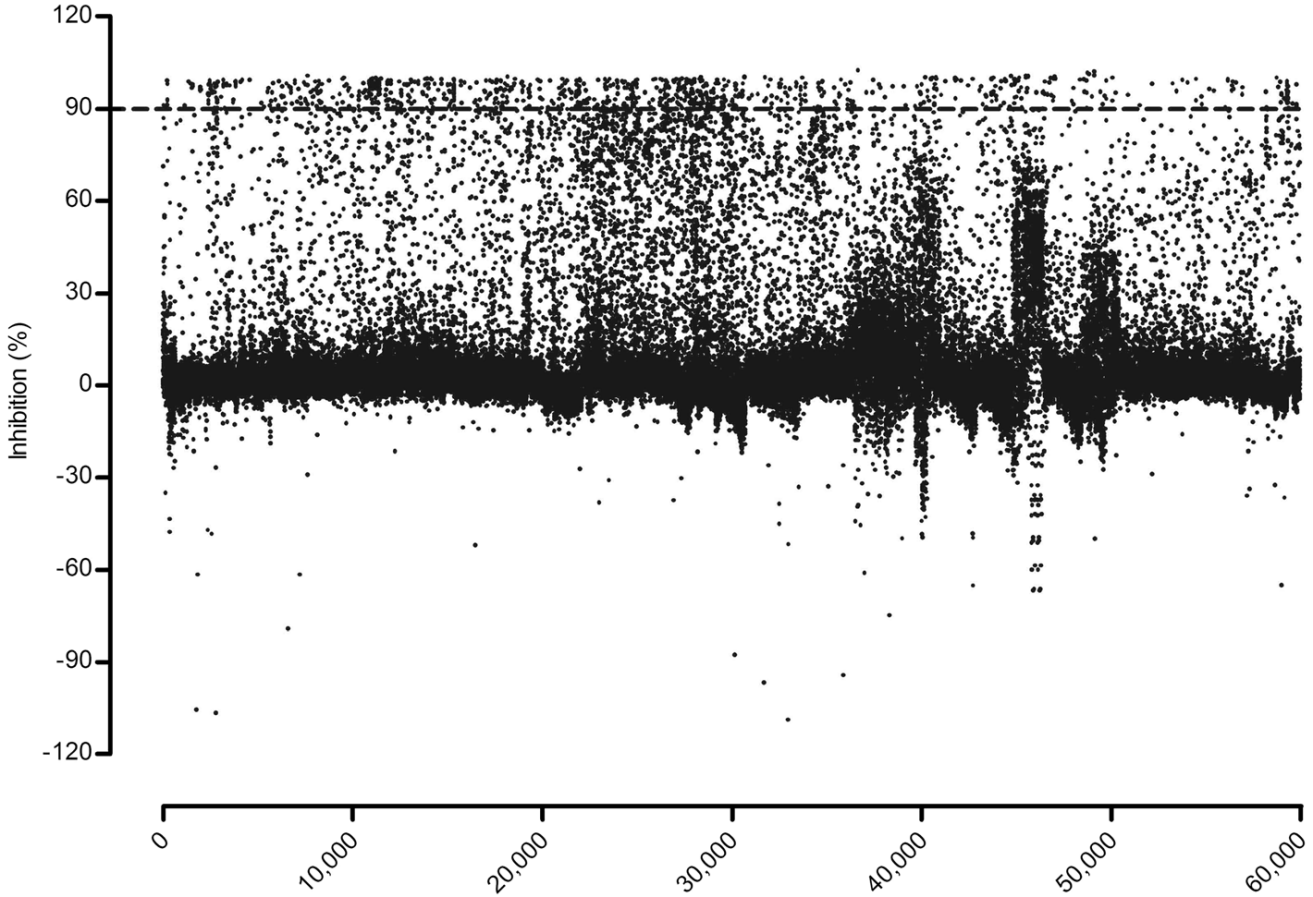
Primary HTS experiment. A collection of 59,360 compounds were screened using the optimized endpoint SmTGR fluorescence assay at an average final concentration of 10 μM for each compound. Sixteen positive (30 μM naphthazarin) and negative (DMSO) control reactions were plated to calculate % inhibition. Dashed line indicates 90 % inhibition of the initial threshold for hits

Under the above optimized assay conditions, we observed good dose responses to naphthazarin with an IC_50_ of 1.42 ± 0.08 μM (see Fig. 1a). This value is comparable to that documented in the literature (10 μM) [8]. Naphthazarin at 30 μM produced a full inhibition (100 %; background signal) of SmTGR in this assay (see Fig. 1b), whereas DMSO alone (served as a negative control) did not show any inhibition (0 %) on SmTGR activity (total signal). As a result, the coefficient of variation (CV) values were 3.40 % for total signal and 13.92 % for background signal, respectively. The Z' factor calculated is 0.859 with a signal-to-background ratio (S/B) of 46.76 (see Fig. 1b). These characteristics indicate that the assay system is of high quality and well suited to HTS [14]. Of the 59,360 samples initially screened, 928 hits (1.56 %) showed greater than 90 % inhibition on SmTGR activity (see Fig. 2). Secondary screening confirmed that 74 (0.12 %) of the above hits displayed consistent inhibitory effects on SmTGR with IC_50_ values ranging between 3 nM and 50 μM (see Table 1).

**Table 1:** Structures of the confirmed hits and their inhibition of SmTGR and cidal activities against worms

Compound	Structure	IC_50_ (μM)^a^	Efficacy (%)^a^	Larva^b^(μM)	Adult^c^(μM)
**WNN0724-H007**		**0.424 ± 0.106**	**98.57 ± 1.36**	**50**	–^e^
**WNN0809-E009**		**3.848 ± 0.311**	**99.60 ± 0.26**	**n.a.** ^**d**^	–
**WNN0256-E003**		**4.476 ± 0.905**	**98.99 ± 0.45**	**n.a.**	–
WNN0434-C002		1.448 ± 0.141	98.48 ± 1.48	25	–
WNN1041-F010		3.181 ± 0.636	97.34 ± 3.30	n.a.	–
WNN0328-C003		5.362 ± 0.453	99.41 ± 0.09	n.a.	–
WNN0625-C003		2.981 ± 0.438	98.69 ± 1.42	n.a.	–
WNN1087-E003		13.21 ± 1.775	95.92 ± 0.12	n.a.	–
**WNN0397-C010**		**0.862 ± 0.120**	**95.55 ± 2.02**	**3.125**	**n.a.**
**WNN0042-E008**		**1.614 ± 0.375**	**94.31 ± 1.53**	**n.a.**	–
WNN0929-D011		0.957 ± 0.232	97.08 ± 1.53	50	–
WNN0929-D007		3.576 ± 0.417	97.86 ± 0.21	n.a.	–
**WNN0464-C005**		**1.038 ± 0.198**	**98.23 ± 2.04**	**25**	–
**WNN0464-F002**		**1.667 ± 0.325**	**97.51 ± 0.75**	**12.5**	–
WNN0474-E006		2.410 ± 0.438	97.22 ± 0.81	n.a.	–
WNN0366-E010		2.605 ± 0.544	73.26 ± 1.61	6.25	–
WNN0544-A007		5.595 ± 0.431	98.62 ± 1.50	n.a.	–
WNN0912-G011		6.633 ± 1.209	96.87 ± 2.64	n.a.	–
**WNN0197-D004**		**3.152 ± 0.509**	**96.75 ± 0.68**	**3.125**	**5**
**WNN0429-D003**		**7.243 ± 0.530**	**98.57 ± 0.63**	**50**	–
**WNN0446-H009**		**6.290 ± 0.177**	**85.95 ± 2.33**	**50**	–
**WNN0194-D004**		**3.238 ± 0.481**	**98.65 ± 1.37**	**50**	–
**WNN0373-D002**		**3.352 ± 0.481**	**82.32 ± 1.51**	**6.25**	–
WNN0464-C004		3.029 ± 0.410	95.73 ± 0.86	6.25	–
WNN0609-E009		7.662 ± 0.870	82.93 ± 1.20	3.125	n.a.
WNN0192-H003		4.900 ± 0.955	94.49 ± 1.31	n.a.	–
**WNN0029-D010**		**7.919 ± 0.488**	**97.19 ± 0.74**	**n.a.**	–
**WNN0029-D009**		**11.89 ± 0.474**	**92.95 ± 0.40**	**n.a.**	–
WNN0224-E010		11.62 ± 0.346	99.21 ± 0.21	50	–
WNN0113-H010		12.78 ± 0.841	92.49 ± 1.05	n.a.	–
WNN0113-H004		9.286 ± 0.622	77.86 ± 2.78	n.a.	–
WNN0113-H003		13.08 ± 0.643	74.21 ± 1.41	n.a.	–
**WNN0009-G004**		**2.595 ± 0.233**	**98.03 ± 1.12**	**12.5**	–
**WNN0341-B004**		**6.076 ± 0.849**	**98.97 ± 0.87**	**n.a.**	–
**WNN0287-H004**		**3.257 ± 0.792**	**91.03 ± 0.45**	**25**	–
WNN0189-G005		15.73 ± 1.351	77.53 ± 0.99	n.a.	–
WNN0621-C011		8.643 ± 0.912	78.09 ± 2.19	n.a.	–
WNN0367-F009		0.612 ± 0.057	98.35 ± 1.32	25	–
**WNN0809-C005**		**17.63 ± 0.863**	**99.20 ± 0.62**	**6.25**	–
**WNN0009-H010**		**28.61 ± 1.131**	**96.77 ± 0.49**	**50**	–
**WNN0409-G007**		**3.548 ± 0.064**	**98.56 ± 1.56**	**n.a.**	–
WNN0197-G002		5.438 ± 0.552	98.09 ± 1.56	3.125	10
WNN0009-A002		14.47 ± 0.990	82.47 ± 1.70	50	–
WNN0925-B011		1.352 ± 0.123	94.87 ± 0.19	n.a.	–
**WNN0029-C005**		**0.003 ± 0.001**	**97.40 ± 1.50**	**12.5**	–
**WNN0726-A010**		**2.614 ± 0.262**	**98.70 ± 1.63**	**50**	–
**WNN0463-G011**		**7.171 ± 0.226**	**95.20 ± 2.54**	**n.a.**	–
WNN0335-G006		13.05 ± 0.028	94.88 ± 1.48	12.5	–
WNN0961-C005		2.643 ± 0.276	95.77 ± 1.08	n.a.	–
**WNN0374-G010**		**4.995 ± 0.290**	**72.99 ± 3.01**	**50**	–
**WNN0304-A007**		**2.886 ± 0.976**	**97.56 ± 2.98**	**50**	–
WNN0826-B004		2.214 ± 0.799	93.88 ± 0.17	25	–
WNN0893-H007		5.019 ± 0.212	95.89 ± 1.26	n.a.	–
WNN0196-F005		6.719 ± 0.233	73.87 ± 0.93	50	–
WNN0493-F008		6.305 ± 0.523	78.85 ± 2.35	3.125	10
WNN0395-A008		7.871 ± 0.728	98.51 ± 1.39	n.a.	–
WNN1018-E008		11.98 ± 0.898	95.62 ± 2.29	n.a.	–
WNN1177-H006		0.831 ± 0.113	98.02 ± 1.44	n.a.	–
WNN0263-A002		16.14 ± 0.884	98.88 ± 0.79	6.25	–
WNN0035-G011		18.23 ± 1.591	96.60 ± 0.67	12.5	–
WNN1177-F009		1.576 ± 0.304	97.00 ± 1.41	n.a.	–
WNN0572-G004		2.119 ± 0.502	98.46 ± 1.61	50	–
WNN0493-H007		3.057 ± 0.495	98.71 ± 0.83	n.a.	–
WNN0826-A003		2.862 ± 0.163	97.36 ± 1.75	50	–
WNN0307-H007		7.348 ± 0.219	98.44 ± 0.80	50	–
WNN0045-G003		9.057 ± 1.117	98.78 ± 0.76	12.5	–
WNN0004-A011		0.004 ± 0.001	95.89 ± 4.91	50	–
WNN0058-C010		0.562 ± 0.071	77.47 ± 1.08	50	–
WNN0433-C010		1.938 ± 0.431	85.21 ± 2.02	n.a.	–
WNN0151-C005		3.286 ± 0.240	97.06 ± 1.17	n.a.	–
WNN0646-C002		14.78 ± 1.619	90.0 ± 1.379	12.5	–
WNN0040-H011		46.90 ± 5.544	96.74 ± 0.72	n.a.	–
WNN0341-C002		4.395 ± 0.403	90.48 ± 1.06	n.a.	–
WNN0826-A007		0.205 ± 0.148	98.36 ± 1.29	n.a.	–

^a^The values shown are means ± SEM of at least three independent experiments. ^b^Compounds were tested against larval worms at 50, 25, 12.5, 6.25, and 3.125 μM. The lowest concentration resulting in 100 % larval death at 48 hours is indicated. ^c^Compounds were tested against adult worms at 10 and 5 μM. The lowest concentration resulting in 100 % worm death on day seven is indicated

^d^n.a.: no activity; ^e^-: not tested

Bold phase indicates compounds in the same scaffold

The 74 confirmed hits can be divided into 17 scaffolds according to their different structure characteristics:

(1) 3a,4,7,7a-tetrahydro-1H-4,7-methanoisoindole-1,3(2H)-dione (e.g., WNN0256-E003) and 3a,4,7,7a-tetrahydro-1H-4,7-epoxyisoindole-1,3(2H)-dione (e.g., WNN0724-H007 and WNN0809-E009); (2) 1H-pyrrole-2,5-dione (e.g., WNN0434-C002, WNN1041-F010, WNN0328-C003, and WNN1087-E003) and its reductive product pyrrolidine-2,5-dione (e.g., WNN0625-C003); (3) 5-nitro-2-(phenylsulfonyl)pyridine (e.g., WNN0397-C010 and WNN0042-E008); (4) ((hydrazono)methyl)phenol (e.g., WNN0929-D011 and WNN0929-D007); (5) 5-(5-nitrothiazol-2-yl)thio)-4H-1,2,4-triazol-3-ol (e.g., WNN0464-C005 and WNN0464-F002); (6) benzoquinone (e.g., WNN0474-E006, WNN0366-E010, WNN0544-A007, and WNN0912-G011); (7) 5-bromo-3,4-dinitrothiophen-2-amine (e.g., WNN0197-D004, WNN0429-D003, and WNN0446-H009) and thiophene derivatives (e.g., WNN0194-D004 and WNN0373-D002); (8) 3-thio-4H-1,2,4-triazole (e.g., WNN0464-C004 and WNN0609-E009) and 2,5-dithio-1,3,4-thiadiazole (e.g., WNN0192-H003); (9) 2-phenyl-2,3-dihydro-1H-naphtho[1,2-e][1,3]oxazine (e.g., WNN0029-D010 and WNN0029-D009); (10) benzo[d][1,3]oxathiol-2-one (e.g., WNN0113-H010, WNN0113-H004, and WNN0113-H003) and its condensation product (e.g., WNN0224-E010); (11) multi-halogen substituted benzene or cyclohexane (e.g., WNN0009-G004, WNN0341-B004, and WNN0287-H004); (12) phenylsulfonyl (e.g., WNN0621-C011 and WNN0367-F009) or pyridinylsulfonyl derivative (e.g., WNN0189-G005); (13) 5-membered heterocycles substituted with nitro or methylsulfonyl group (e.g., WNN0809-C005, WNN0009-H010 and WNN0409-G007); (14) nitro substituted benzo[b]thiophene derivatives (WNN0197-G002, WNN0009-A002, and WNN0925-B011); (15) benzofuran (e.g., WNN0463-G011) and 2,3-dihydrobenzofuran (e.g., WNN0029-C005 and WNN0726-A010); (16) benzo[c][1,2,5]oxadiazole (e.g., WNN0335-G006 and WNN0961-C005); and (17) quinolin-2(1H)-one (e.g., WNN0374-G010 and WNN0304-A007).

A limited structure-activity relationship (SAR) could be inferred from some of the scaffolds: for instance, in scaffold (5) (WNN0464-C005 versus WNN0464-F002), N-substituent including cycloalkyl and alkyl chain had no obvious difference for TGR inhibition, with WNN0464-F002 exhibiting better worm-killing activity *ex vivo*; in scaffold (7) (WNN0197-D004 versus WNN0429-D003), substituents including ethyl and benzyl on the amino group also showed no obvious difference for TGR inhibition, however, WNN0197-D004 was active *ex vivo*. Among all these hits, furoxan (WNN0809-C005) and benzofuroxan (WNN0335-G006) were well-studied structural motifs as nitric oxide (NO) donating compounds capable of controlling schistosomiasis [11, 18]. The mechanism involved in this action includes nucleophilic attack by the sulfhydryl moiety of a cysteine residue or by selenocysteine at either three or four-position of the oxadiazole, and subsequent rearrangement to release the nitroxyl anion (NO^−^). Further SAR analysis showed that the presence of electron withdrawing groups at three-position generally increased its capacity. Fruttero and co-workers reported that furoxans could inhibit the activity of P-glycoprotein, MRP1, and MRP3 (multidrug resistance-associated proteins) transporters in different types of cells, which were considered as potentially attractive targets for the development of new therapeutic agents against schistosomal infection [19].

### *Ex vivo* activity against parasites

The 74 confirmed hits were screened against cultured, larval parasites at 50 μM (see Table 1). We found that 53 % (39/74) of the compounds had cidal activity at 48 h. They were subsequently tested against larva at 25, 12.5, 6.25, and 3.125 μM (see Table 1). Of them, five were found to kill 100 % of larva at 48 h at 3.125 μM (see Table 1). These five hits were further tested *ex vivo* against adult worms, with two killing adult worms at 10 μM (WNN0197-G002 and WNN0493-F008) and one killing adult worms at 5 μM (WNN0197-D004; see Table 1).

## Discussion

To date, PZQ is the only drug used to treat the disease, and with its increased use the probability of developing resistance has grown significantly. Therefore, it is important to identify new drugs to replace PZQ or, preferably, to be used in combination with PZQ to prevent resistance from developing. It is known that *S. mansoni* survives in humans partly because of the existence of a set of antioxidant enzymes that continuously degrade reactive oxygen species produced by the host. A key element of this defense system is TGR, a parasite-specific enzyme combining the functions of three human counterparts: glutathione reductase, thioredoxin reductase, and glutaredoxin. Although a recent study reported that RNAi helped validate novel and putative drug targets for schistosomiasis [20], TGR remains an attractive one and has been subject to HTS studies using different assay systems [13, 21]. In this study, we used recombinant SmTGR and adapted a novel coupled assay to measure TGR activity alone, which in turn was optimized for HTS [14]. This validation process was successful as demonstrated by a comparable IC_50_ value measured for the positive control, naphthazarin (see Fig. 1a), and other high quality assay parameters such as CV, Z' factor, and S/B (see Fig. 1b).

In the primary screening, a total of 59,360 synthetic compounds were screened and 928 initial hits were identified (1.56 %) using 90 % inhibition on SmTGR activity as a cut-off point. The initial hits were further characterized in dose–response experiments, of which 74 compounds displayed consistent inhibitory properties. When assessed for *ex vivo* activity against parasites, 39 hits showed cidal effects in 48 h, and five of them displayed 100 % killing at 3.125 μM; of these, two killed adult worms at 10 μM (WNN0197-G002 and WNN0493-F008) and one at 5 μM (WNN0197-D004), respectively. The bioactivity data shown in Table 1 indicate that there is no real trend or relationship between TGR inhibition and worm-killing activity. This phenomenon is not surprising as drug uptake by worms is usually the limiting factor in compound activity. In previous studies, most worm-killing activity was seen only at high nanomolar or low micromolar concentrations, e.g., 0.3–1 μM for PZQ [22]. This may be due to slow metabolism of worms cultured *ex vivo*, low compound uptake by worms, or many other factors. Three confirmed hits (WNN0197-G002, WNN0493-F008, and WNN0197-D004) were more active against worms than against TGR suggesting they hit other targets. TGR inhibitors are frequently electrophiles [10] and some of the more potent TGR inhibitors identified in this study have strong electrophilic centers (e.g., nitro, sulfonyl, methylsulfonyl, *etc*.), and might react with thiols and/or other components in the media thereby reducing their ability to kill worms.

## Conclusion

Clearly, use of TGR as a molecular target to identify chemical compounds with inhibitory property is a viable approach in the development of therapeutic agents against schistosomiasis. The HTS system optimized in this study is both robust and easy to perform; it provides an alternative to other screening assays such as NADPH fluorescence [13] and DTNB (5,5’ dithiobis(2-nitrobenzoic acid) reduction [21] focusing on this enzyme. Identification of hits with structures similar to those previously shown to kill the parasite further supports the assay validity. Of the 74 hits discovered, the large variety of scaffolds offers adequate chemical space for lead generation and other follow-up investigations, though some of them may represent false positives that require secondary verification of their functionality. In this regard, the present study will certainly attract attention from scientists who are engaged in schistosomiasis research.

## Abbreviations

BSA: Bovine serum albumin
CV: Coefficient of variation
DMSO: Dimethyl sulfoxide
EDTA: Ethylenediaminetetraacetic acid
GSH: Reduced glutathione
GSSG: Oxidized glutathione
HTS: High-throughput screening
NADPH: β-nicotinamide adenine dinucleotide 2'-phosphate reduced tetrasodium salt hydrate
MRP: Multidrug resistance-associated protein
PZQ: Praziquantel
SAR: Structure-activity relationship
S/B: Signal-to-background ratio
SEM: Standard error of the mean
SmTGR: *Schistosoma mansoni* thioredoxin glutathione reductase
TGR: Thioredoxin glutathione reductase
